# Is game-based therapy effective for treating cognitive deficits in adults with schizophrenia? Evidence from a randomized controlled trial

**DOI:** 10.1038/s41398-024-02920-0

**Published:** 2024-07-16

**Authors:** Junkai Wang, Jie Zhang, Peng Xu, Tianyi Qian, Shuping Tan, Peipeng Liang

**Affiliations:** 1 School of Psychology, Capital Normal University, Beijing, China; 2 Beijing Key Laboratory of Learning and Cognition, Beijing, China; 3 Department of Radiology, Aerospace Center Hospital, Beijing, China; 4 Psychiatry Research Center, Beijing Huilongguan Hospital, Peking University Huilongguan Clinical Medical School, Beijing, China; 5 Tencent Healthcare, Shenzhen, China

**Keywords:** Schizophrenia, Human behaviour

## Abstract

Cognitive deficits in schizophrenia are a major contributor to poor functional outcomes and everyday functioning, making them a promising therapeutic target. Recent years have witnessed a dramatic increase in the use of digital interventions, such as game-based therapy, targeting various domains of cognition to treat mental disorders. Game-based digital interventions have been suggested to have therapeutic value in health care for people with schizophrenia. To support this idea, a novel, online training program (Komori Life) that targets cognitive deficits in schizophrenia was tested for feasibility of use and initial efficiency. Inpatients with schizophrenia were randomized to complete 20 sessions of either Komori Life (*N* = 40 completers) or treatment as usual (*N* = 40 completers). Cognitive and clinical assessments were performed at enrollment and after completion of the training intervention for all patients. In addition, 32 healthy volunteers were recruited as controls, and an eye-tracking paradigm was employed to assess attentional biases to emotional information before and after game intervention for all subjects. The results showed that there were no group differences in cognitive or clinical assessments at baseline between the two patient groups. After game training, there were still no group × time interactions on cognitive or clinical assessment scores. Regarding eye movement measurements, both patient groups showed increased attention to threatening stimuli compared to healthy controls in terms of attentional maintenance at baseline. After game training, the game training group revealed greater improvement in attentional bias towards threatening scenes (decreased percentage of total duration and percentage of total fixations towards threatening stimuli) relative to the treatment as usual group. Moreover, our results partially indicated that training effectiveness was associated with cognitive improvement and that heightened attentional maintenance to threats was associated with worse cognitive performance. This study provides initial evidence that a remote, online cognitive training program is feasible and effective in improving cognitive function in schizophrenia. This form of training may serve as a complementary therapy to existing psychiatric care. Clinical trial registration: the trial is registered at http://www.chictr.org.cn, identifier ChiCTR2100048403.

## Introduction

Schizophrenia (SZ) is a complex, severe mental disorder characterized by a wide range of psychotic symptoms, including positive symptoms (such as delusions and hallucinations) and negative symptoms (such as emotional withdrawal, anhedonia and amotivation) [1, 2], and is associated with considerable deficits in all core domains of higher-order cognitive functions [3, 4]. Cognitive impairment is now a well-documented feature of schizophrenia and affects several cognitive domains, including attention, verbal learning, long-term memory, and executive function [3, 4], and core domains of social cognition, including affect perception, social cue perception, theory of mind and attributional style [5–8]. Importantly, there is increasing evidence that cognitive deficits in schizophrenia are a major contributor to poor social and occupational functioning, making them a promising therapeutic target [9, 10]. Currently, a variety of first-generation and second-generation antipsychotics, which are the most common treatments for schizophrenia, are effective for psychotic symptoms [11]. However, the effects of pharmacological treatments on cognitive impairment have thus far demonstrated only limited efficacy [11–13]. Therefore, new alternative methods are needed to alleviate cognitive impairments and improve patients’ everyday functioning.

In recent years, cognitive remediation therapy has produced promising findings, and such studies have shown a modest effect size for global cognitive improvement among people diagnosed with schizophrenia [14, 15]. Despite the encouraging results of cognitive remediation therapy, these interventions are far from becoming the standard of care and are potentially limited by their scalability and high dropout rate [16, 17]. More specifically, such interventions require highly trained professionals, long treatment durations, and regular hospital visits, and a lack of motivation among schizophrenia patients is more likely to lead to dropout from treatments [16]. Therefore, interesting and attractive interventions are urgently needed to improve cognitive impairments in schizophrenia. Computerized interventions, including computer games [18, 19], virtual reality [20, 21], and serious games [22, 23], which are created for cognitive training, can overcome those limitations. Different computer-based interventions have been designed to target various domains of cognition and have proven to be feasible and potentially beneficial in related cognitive abilities [24]. Nevertheless, therapeutic game interventions represent an emerging field in which constant updates are needed, and few computerized trainings have been broadly administered to improve higher-level cognition in schizophrenia [24]. Here, this study examined the feasibility and initial efficacy of a novel, online game intervention (Komori Life by Tencent) among adults with schizophrenia. Komori Life is a social/life simulation game and consists of multiple training exercises targeting various domains of cognition that range from general cognitive abilities to social interaction. The Komori Life exercises are adaptive based on individual performance that progressively involves more complex tasks.

Although game interventions have shown potentially beneficial effects on the improvement of cognition in schizophrenia, recent methodologically rigorous trials and systematic reviews have reported discouraging results [25, 26]. One possible reason for the negative findings in previous studies may be the lack of objective methods to evaluate intervention outcomes. Common assessments of cognitive function and psychotic symptoms combined with objective evaluation methods might be able to sensitively assess the efficacy of cognitive intervention. Previous studies have revealed atypical attentional patterns to emotional information among patients with schizophrenia [27]. More specifically, attentional biases towards emotionally relevant content, such as threatening information, have been suggested to be involved in the etiology of a wide range of schizophrenia symptoms, which might be a sensitive marker for evaluating the severity of schizophrenia [28–30]. To obtain a detailed measurement of attentional processing, eye tracking is considered an excellent technique for capturing cognitive processes during visual tasks [31]. Eye-tracking paradigms have been repeatedly used to examine attentional biases towards threatening information among individuals with schizophrenia, and studies have consistently suggested that threat-related attentional bias may represent an underlying psychological mechanism of schizophrenia and that related eye-tracking measures are associated with the severity of schizophrenia [32–34].

To summarize, a randomized controlled trial was conducted to examine the feasibility and initial efficacy of Komori Life training using tablet computers. An eye-tracking paradigm was employed to assess attentional biases towards emotional information before and after the game intervention. The hypothesis of this study was that the experimental group participants (Komori Life training) would show greater improvement in cognitive function relative to the treatment as usual group, according to ameliorating atypical attentional patterns to emotional information.

## Methods

The trial was a parallel group, efficacy trial of game-based therapy among chronic schizophrenia patients. The trial is registered at http://www.chictr.org.cn, identifier ChiCTR2100048403.

### Participants

Eighty clinically stable patients with chronic schizophrenia were recruited from Beijing Huilongguan Hospital. Thirty-two age-matched healthy controls (HCs) who were recruited through advertisement also participated in this study. The patients were required to meet the Diagnostic and Statistical Manual of Mental Disorders-V (DSM-V) criteria for schizophrenia or schizoaffective disorder based on interviews and a review of their clinical histories. Other inclusion criteria included age between 16 and 45 years, more than 6 years of education, moderate negative symptoms using the Positive and Negative Syndrome Scale (PANSS) [35], and relative clinical stability. The exclusion criteria for all subjects included cognitive impairment caused by head trauma or cranial surgery, neurological deficiencies such as visual or hearing loss, alcohol or substance abuse/dependence within the previous 6 months, and game addiction (playing games for ≥3 h/day for the last 6 months). All volunteers received financial compensation for their participation. The authors assert that all procedures contributing to this work comply with the ethical standards of the relevant national and institutional committees on human experimentation and with the Helsinki Declaration of 1975, as revised in 2008. The experimental procedure was approved by the Institutional Review Boards (IRBs) of Beijing Huilongguan Hospital, Peking University. Written informed consent was obtained from all volunteers after the study had been fully explained.

### Study procedures

Eligible SZ patients completed baseline assessments, including cognitive, clinical, and functional tests and eye-tracking tasks, in the ward. Then, these patients were randomly assigned to either game training (GT, Komori Life (see below) and routine treatment) or treatment as usual (TAU, routine treatment) training conditions (also see the CONSORT checklist). All subjects were grouped into blocks of 2 showing similar demographic characteristics (age, sex and education), and each of the 2 patients in a block was then randomly assigned to a different group to minimize the imbalance. Participants in the GT group were loaned tablet computers, given logins and instructed to complete their training intervention 5 times/week, lasting for 60 min each time over 4 weeks. After 4 weeks, all patients were asked to complete the posttraining assessment battery (same tests and task as the baseline assessments). During the training, participants interacted with staff who supervised the patients if they indicated difficulty in completing training. Participants in the TAU group as well as those in the GT group participated in their daily rehabilitative activities. Following consent and initial screening, HCs completed the eye-tracking task only.

### The Komori Life training program

This training program was modified from Komori Life (https://komori.qq.com), which was deployed on an online, browser-playable platform by Tencent. Komori Life is a social/life and farming simulation game. In the game, the user can choose to play the role of a student or an office worker who has just moved to a small town in the Japanese countryside. The user goal is, starting from scratch, to decorate the house, fix up garden patches and plant fruit trees or vegetables, catch animals, cut down trees, extract useful minerals, cook food and get to know all the inhabitants in the town (see online Supplementary Material Fig. S1 and Supplementary Table 15). In this process, participants complete exercises on all of the cognitive domains related to attention, memory, executive function and social cognition. This program begins with lower cognitive demands and progressively advances to more complex exercises. Progression is guided through the training interactively, and feedback is given when completing a level in the game. Participants completed unique games during every training session for ~60 min per session. Game-related measures include the game grades, total playing time, total game playing behaviors (planting, cooking, decorating, hunting, felling and mining) and degree of activity.

### Assessments

Cognitive and clinical assessments were performed at baseline and after completion of the training intervention among all participants with SZ. All neuropsychological tests were conducted by graduate psychologists working in hospitals.

The positive and negative symptoms were assessed by using PANSS [35] and the Brief Negative Symptom Scale [36]. Social function, quality of life and pleasure experience were measured with the Personal and Social Performance Scale (PSP), Self-Esteem Scale [37], Schizophrenia Quality of Life Scale and Temporal Experience of Pleasure Scale [38, 39]. Cognitive function was assessed with the Measurement and Treatment Research to Improve Cognition in Schizophrenia (MATRICS) consensus cognitive battery according to 7 cognitive domains of the MCCB, including speed of processing, attention/vigilance, verbal learning, visual learning, reasoning and problem solving and social cognition [40].

### Eye-tracking task

A free-viewing task with emotional scenes was performed at baseline and after completion of the training intervention among all SZ participants as well as among HCs. The full description of this task has been published elsewhere, and most relevant details of task procedures are described below [32].

#### Stimuli

The stimuli included 80 images selected from the International Affective Picture System [41]. The stimuli were categorized as sad, happy, threatening, or neutral following a pilot study [42]. A total of 12 sad, 12 happy, 12 threatening and 12 neutral target social images as well as 32 neutral control images were chosen. The sad, happy and threatening target images represented people showing different emotions under different situations. Neutral target images represented people in nonemotional activities. The neutral control images represented nonliving objects [43]. Threatening images had the greatest arousal rating, and neutral images had the lowest arousal rating among the four categories of images. The sad and happy images did not differ from each other in arousal. The happy and neutral images had higher valence ratings than the other categories of images, whereas the sad and threatening images did not differ from each other in valence ratings. The images did not differ in visual complexity, which was transferred in JPEG compressed file format [44].

#### Free-viewing task

**Fig. 1:**
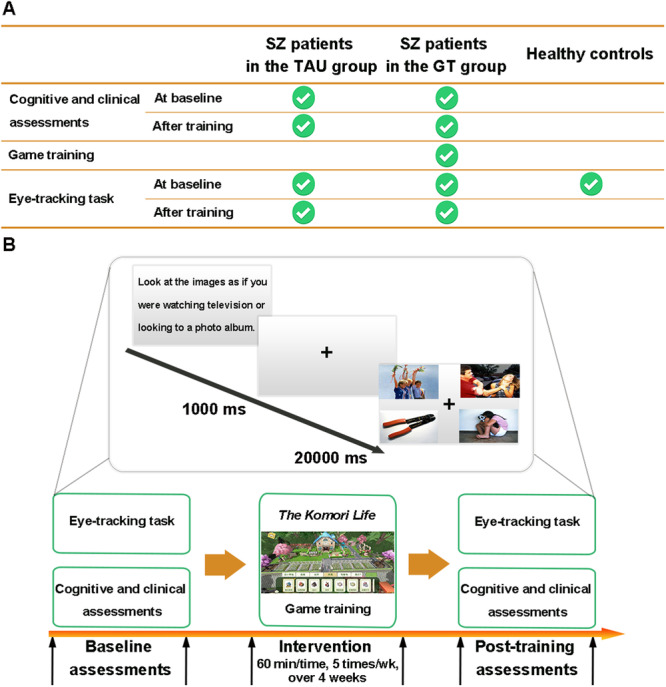
Study procedures of the study. **A** SZ patients completed baseline assessments and the post-training assessment battery (same tests and task with baseline assessments) during the procedure and the HC completed the eye-tracking task only. **B** The details of the whole procedures and free-viewing task. GT game training, TAU treatment as Usual, SZ schizophrenia.

A total of twenty trials (12 study trials and 8 filler trials) containing four images were simultaneously presented. Each trial began with a 1000-ms centrally displayed fixation cross. Then, the trial was presented for 20 s. Twelve study trials contained four images on each slide, and each image was selected from the following four categories: sad, happy, threatening, or neutral target social images. Eight filler trials with four neutral control images were displayed to obscure the nature of the task. For each study trial, the position of each image was randomly selected, with the constraint that each valence must occur in each of the four positions three times across the 12 trials. The presentation order of the trials was also randomized across the subjects [32, 43], see Fig. 1.

#### Apparatus

A remote eye-tracking binocular system (aSee Pro, 7invensun, Beijing, China) was employed to measure the subjects’ eye movements, which allowed a free range of head movements. The sampling rate of the eye positions was 135 Hz. Subjects were seated 65 cm away from the screen. Camera adjustments were made to best capture the subjects’ eyes.

#### Procedure

After completing the baseline assessments, participants were tested individually in a silent room. The experimental session began once the calibration was accepted (the average error was less than 1.5° of the visual angle for each calibration point). Then, participants were instructed via the computer screen with the following content: “Look freely at the images as if you were watching television or looking at a photo album”. The experimenter was located in the same room and monitored the whole process. The size of the pictures was 10.95° (wide) × 6.20° (high), and the distance between the pictures was 5.44° (horizontal axis) and 3.06° (vertical axis).

### Data analysis

To ensure a balance between all baseline measures in each SZ patient group, eye movement measures and cognitive and clinical assessments were compared. Continuous data are presented as the means and standard deviations. Group differences in demographic measures were tested using one-way analysis of variance (one-way ANOVA), and chi-square analyses or Fisher’s exact tests were used for quantitative and qualitative variables. Group differences in cognitive and clinical measures at baseline between the two SZ patient groups were tested using the independent sample *t-*test. Group differences in cognitive and clinical measures after training were analyzed in separate repeated-measures ANOVAs with group (the GT group and the TAU group) as a between-subject factor and before and after assessments as a within-subject factor.

For eye movement data, the areas of interest were identified for each study trial and corresponded to the total area for each of the four images. Each area of interest for each target image was added 2 mm to both the up and down, left and right. A total of 5 measurements were computed to evaluate attention across different target images. Two measurements assessed allocation of attention were as follows: (1) the percentage of total fixations (i.e., percentage of times that each subject fixated, and refixated, on a particular target image); and (2) the percentage of total duration (i.e., percentage of fixation time attending to each target image). In addition, three measurements were employed to assess subsequent attentional engagement as follows: (1) first-pass fixations (i.e., the sum of fixations made on the image when looking at it for the first time, before fixating away from it); (2) percentage of first fixation (i.e., percentage of times that the first fixation lands on the image); and (3) gaze duration (sum of fixation duration made on the target image when looking at it for the first time) [32, 33]. All measures were averaged across the trials. Each eye movement measure at baseline was analyzed in two-way ANOVAs with group (the GT group, the TAU group and the HC group) as a between-subject factor and valence (sad, happy, threatening and neutral) as a within-subject factor. For eye movement measures after training, group differences were analyzed in repeated-measures ANOVAs with group (the GT group and the TAU group) as a between-subject factor and (before and after assessments) as a within-subject factor. The aforementioned ANOVAs were also analyzed with the participant’s sex, age, and years of education as covariates. We were only interested in the interaction effect of group by valence or group by time. If the interaction effect was significant, simple effects tests were performed, which were corrected for multiple comparisons by Bonferroni comparisons.

Additionally, correlational analyses were performed to characterize the relationships among game-related measures, cognitive and clinical measure changes before and after training, and eye movement measure changes before and after training in the two SZ patient groups separately. Bonferroni multiple comparisons were employed to control for type-I errors due to multiple testing. SPSS (version 21.0, IBM) was utilized for statistical analyses. A value of *p* < 0.05 was considered statistically significant.

## Results

### Participant and baseline characteristics

**Fig. 2:**
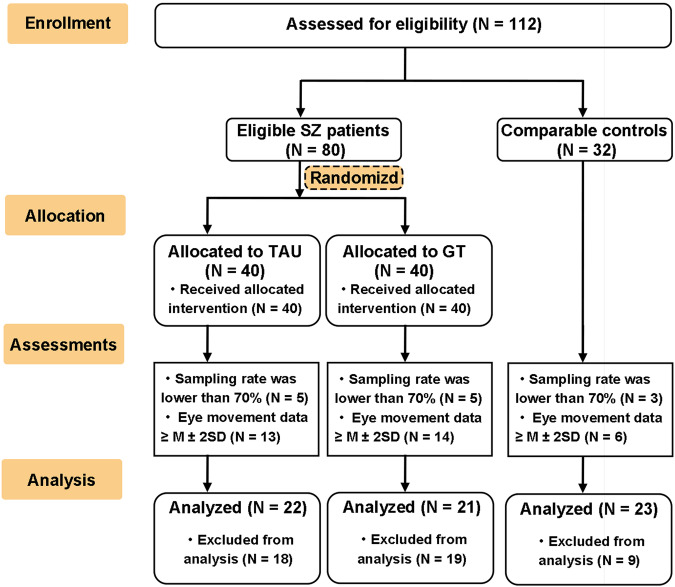
A CONSORT diagram for the clinical trial. GT game training, TAU treatment as Usual, SZ schizophrenia.

Recruitment took place from December 2021, and the posttraining assessment was completed in January 2022. The flow diagram of enrollment and participation in the trial is shown in Fig. 2. A total of 112 participants comprising 80 SZ patients and 32 HCs who were assessed for eligibility entered this study. Forty SZ patients were allocated to the GT group, and the other half of the patients were allocated to the TAU group. All SZ patients and HCs were assessed with an eye-tracking task. Due to the lower gaze samples (the gaze samples were lower than 70% among 13 participants) and outlier data (33 participants’ eye movement data were more than 2 SDs above the normal mean), 21 SZ patients in the GT group (8 males and 13 females), 22 SZ patients in the TAU group (13 males and 9 females) and 23 HCs (5 males and 18 females) entered the final eye movement data analysis. Some SZ patients failed to complete the cognitive and clinical assessments at baseline and posttraining for the following reasons: (1) poor understanding and (2) discharge from the hospital before the posttraining assessments. None of the participants reported any discomfort during the whole experimental period, and none of the assessors observed any adverse reactions among the participants.

Demographic data are listed in Table 1. The two SZ groups and the HC group were well matched for age (*F*(2, 109) = 1.65, *p* = 0.20) and education level (*F*(2, 109) = 0.97, *p* = 0.38), but there was a slight difference in sex (*p* = 0.029) among the three groups. Both patient groups had more females than males relative to the HC group. There were no group differences in age of onset or antipsychotic dose in chlorpromazine equivalents between the two SZ groups.

**Table 1:** Demographic and clinical information of SZ patients and HC.

	GT (*n* = 40)	TAU (*n* = 40)	HC (*n* = 32)	*p* Value
Age, mean (SD), yr	49.6 (9.5)	49.7 (9.6)	45.8 (11.9)	0.20
Sex, males/females	17/23	21/19	7/25	0.029*
Education, mean (SD), yr	12.6 (2.6)	13 (3.5)	11.9 (4.0)	0.38
age of onset (SD), yr	24.9 (8.3)	25.0 (8.4)	/	0.86
Antipsychotic dose^a^	486.5 (248.7)	487.1 (254.3)	/	0.62

One-way ANOVA and Chi-square analyses were applied to test for group differences, statistical significance level was set at *p* < 0.05 (two-tailed). Significant *p* values were in bold.

*GT* game training, *TAU* treatment as Usual, *HC* healthy controls.

*The *p* value was obtained using Chi-square test with multiple comparison.

^a^Calculated as chlorpromazine equivalents.

### Cognitive and clinical assessments at baseline and posttraining

There were also no group differences in cognitive or clinical assessments at baseline between the two SZ groups (see Supplementary Table 1). After Komori Life training, there were still no group × time interactions on PANSS, BNSS, PSP, SES, SQLS, TEPS or MCCB scores (see Supplementary Tables 2 and 12 for actual outcome values).

### Eye movement measurements at baseline

The descriptive data for threatening and happy conditions in the three groups are presented in Supplementary Table 3. All main effects and interactions of ANOVAs for threatening and happy conditions in the three groups are shown in Supplementary Table 5.

#### Percentage of total fixations

**Fig. 3:**
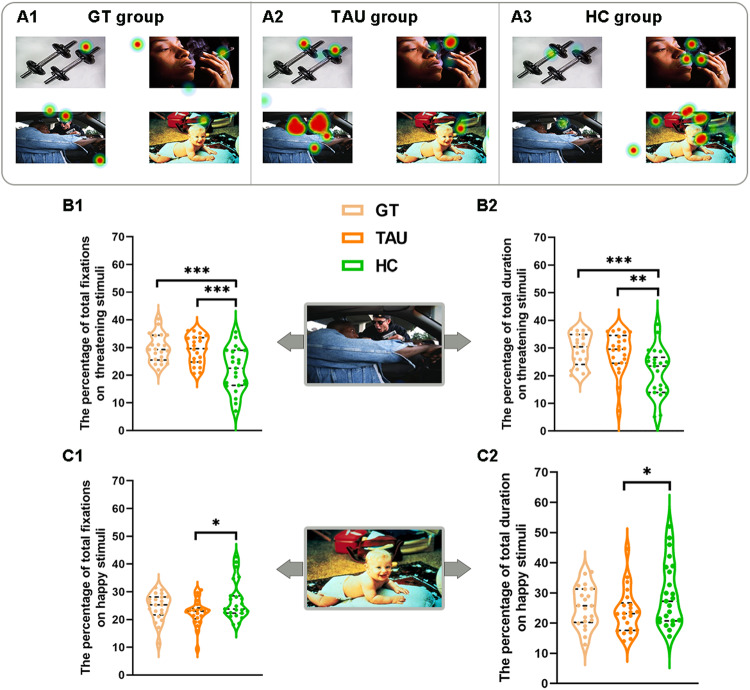
The percentage of total fixations and the percentage of total duration on threatening stimuli and happy stimuli for the three groups at baseline. Representative hotspot map for the GT group (**A1**), the TAU group (**A2**), and the HC group. The ANOVA revealed significantly higher percentage of total fixations to threatening stimuli for the SZ patients than for the HC group (**B1**), and higher percentage of total duration to threatening stimuli for the SZ patients than for the HC group (**B2**). The ANOVA also revealed significantly higher percentage of total fixations on happy stimuli for the HC group than for the TAU group (**C1**), and higher percentage of total duration on happy stimuli for the HC group than for the TAU group (**C2**). *p ≤ 0.05, **p ≤ 0.01, ***p ≤ 0.001. GT game training, TAU treatment as Usual, HC healthy controls.

The ANOVA of the percentage of total fixations revealed an effect of valence (*F*(3, 252) = 27.12, *p* < 0.001) and a significant valence × group interaction (*F*(6, 252) = 5.74, *p* < 0.001) among the SZ patients in the GT group, the SZ patients in the TAU group and the HC group. Simple effects tests showed that the percentage of total fixations to threatening stimuli was higher for the SZ patients in the GT group than for the HC group (*p* < 0.001) and for the SZ patients in the TAU group than for the HC group (*p* = 0.001). Conversely, the percentage of total fixations on happy stimuli was higher for the HC group than for the SZ patients in the TAU group (*p* = 0.021) (see Fig. 3B1, C1). The aforementioned results were replicated after controlling for age, sex and education level (see Supplementary Table 6).

#### Percentage of total duration

Similar to the percentage of total fixations, the ANOVA of the percentage of total duration showed an effect of valence (*F*(3, 252) = 27.23, *p* < 0.001) and a significant valence × group interaction (*F*(6, 252) = 4.94, *p* < 0.001) among the SZ patients in the GT group, SZ patients in the TAU group and the HC group. Simple effects tests revealed that the percentage of total duration to threatening stimuli was higher for the SZ patients in the GT group than for the HC group (*p* = 0.001) and for the SZ patients in the TAU group than for the HC group (*p* = 0.004). Conversely, the percentage of total duration on happy stimuli was higher for the HC group than for the SZ patients in the TAU group (*p* = 0.044), see Fig. 3B2, C2. The aforementioned results were also replicated after controlling for age, sex and education level (see Supplementary Table 6).

No significant valence × group interactions were found for other eye movement measurements, such as first-pass fixations, percentage of first fixation or gaze duration (see Supplementary Tables 5 and 6).

### Eye movement measures after training

The main effects and interactions of ANOVAs for threatening and happy conditions in the two SZ patient groups after training are shown in Supplementary Table 7.

#### Percentage of total fixations

**Fig. 4:**
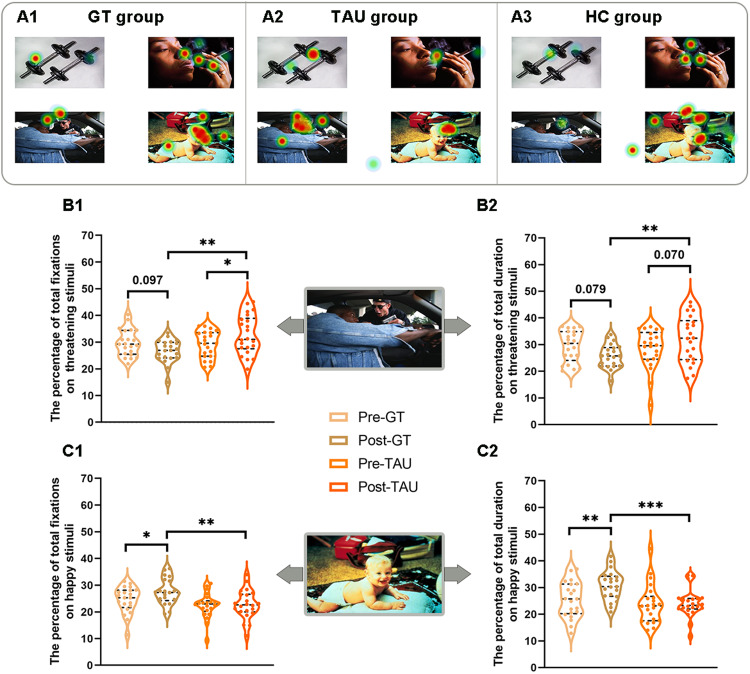
The percentage of total fixations and the percentage of total duration on threatening stimuli and happy stimuli for the patient groups after training. Representative hotspot map for the GT group (**A1**), the TAU group (**A2**), and the HC group. The ANOVA of the percentage of total fixations to threatening stimuli showed an effect of group and a significant time × group interaction between two SZ patient groups (**B1**). The ANOVA of the percentage of total duration on threatening stimuli revealed a significant time × group interaction between two SZ patient groups (**B2**). The ANOVA of the percentage of total fixations to happy stimuli also revealed an effect of group between two SZ patient groups (**C1**). The ANOVA of the percentage of total duration on happy stimuli also revealed an effect of group, an effect of time and a significant time × group interaction between two SZ patient groups (**C2**). *p ≤ 0.05, **p ≤ 0.01, ***p ≤ 0.001. GT game training, TAU treatment as Usual, HC healthy controls.

The ANOVA of the percentage of total fixations to threatening stimuli showed an effect of group (*F*(1, 41) = 6.05, *p* = 0.018) and a significant time × group interaction (*F*(1, 41) = 6.95, *p* = 0.012) between the two SZ patient groups. The ANOVA of the percentage of total fixations to happy stimuli also revealed an effect of group (*F*(1, 41) = 9.06, *p* = 0.004) between the two SZ patient groups. Simple effects tests revealed that the percentage of total fixations to threatening stimuli showed a decreasing trend after game-based therapy compared to baseline value (*p* = 0.097), and the percentage of total fixations to happy stimuli was significantly increased after game-based therapy compared to baseline (*p* = 0.043). However, the percentage of total fixations to threatening stimuli was significantly increased for the SZ patients in the TAU group after routine treatment compared to the baseline value (*p* = 0.049), and there was no difference in the percentage of total fixations to happy stimuli in the TAU group between baseline and after routine treatment. Moreover, the percentage of total fixations to threatening stimuli was higher for the SZ patients in the TAU group than for the SZ patients in the GT group (*p* = 0.002), and the percentage of total fixations to happy stimuli was higher for the SZ patients in the GT group than for the SZ patients in the TAU group (*p* = 0.004). The above differences still existed after controlling for age, sex and education level, suggesting that SZ patients in the GT group had more improvement than the other group (see Fig. 4B1, C1 and Supplementary Table 8).

#### Percentage of total duration

Similarly, ANOVA of the percentage of total duration on threatening stimuli revealed a significant time × group interaction (*F*(1, 41) = 6.69, *p* = 0.013) between the two SZ patient groups. The ANOVA of the percentage of total duration on happy stimuli also revealed an effect of group (*F*(1, 41) = 7.28, *p* = 0.010), an effect of time (*F*(1, 41) = 5.87, *p* = 0.020) and a significant time × group interaction (*F*(1, 41) = 4.81, *p* = 0.034) between the two SZ patient groups. Simple effects tests revealed that the percentage of total duration on threatening stimuli showed a decreasing trend in the GT group after game-based therapy compared to baseline value (*p* = 0.079), and the percentage of total duration on happy stimuli was significantly increased after game-based therapy compared to baseline (*p* = 0.002). However, no differences were found for the percentage of total duration on threatening stimuli or on happy stimuli in the TAU group between baseline and after routine treatment. Moreover, the percentage of total duration on threatening stimuli was higher for the SZ patients in the TAU group than for the SZ patients in the GT group (*p* = 0.004), and the percentage of total duration on happy stimuli was higher for the SZ patients in the GT group than for the SZ patients in the TAU group (*p* < 0.001). The above differences still existed after controlling for age, sex and education level (see Fig. 4B2, C2 and Supplementary Table 8).

No significant time × group interactions were found for the percentage of total fixations to sad or neutral stimuli, the percentage of total duration on sad or neutral stimuli, and other eye movement measurements, such as first-pass fixations, percentage of first fixation or gaze duration (see Supplementary Tables 9–11).

### Correlational analyses

**Fig. 5:**
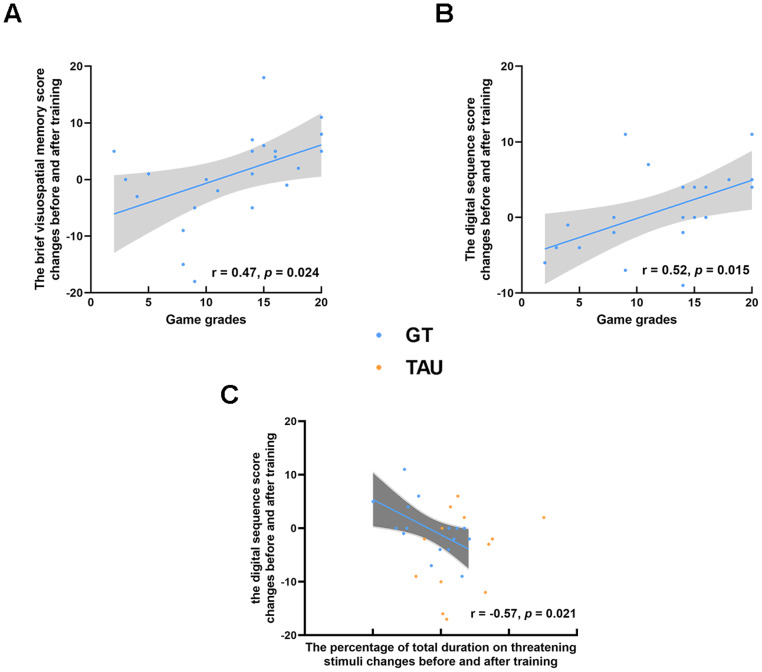
The relationship among game-related measures, cognitive and clinical improvement scores, and eye movement improvement scores in the two SZ patient groups, respectively. Game grades showed positive correlations with the brief visuospatial memory score changes before and after training (**A**) and the digital sequence score changes before and after training (**B**) in the GT group. The percentage of total duration on threatening stimuli changes before and after training showed negative correlations with the digital sequence score changes before and after game training (**C**) in the GT group. GT game training, TAU treatment as Usual.

Finally, correlation coefficients were computed among game-related measures, cognitive and clinical improvement scores (posttraining values-baseline values), and eye movement improvement scores (posttraining values-baseline values) in the two SZ patient groups. For the SZ patients in the GT group, game grades showed positive correlations with the brief visuospatial memory score changes before and after training (*r* = 0.47, *p* = 0.024, uncorrected) and the digital sequence score changes before and after training (*r* = 0.52, *p* = 0.015, uncorrected) (see Fig. 5A, B). The percentage of total duration on threatening stimuli changes before and after training showed negative correlations with the digital sequence score changes before and after game training (*r* = −0.57, *p* = 0.021, uncorrected) (see Fig. 5C). The above significant correlations still existed after controlling for the influence of sex, age, and education level. However, none of these correlation coefficients were significant after correcting for multiple comparisons. Apart from these, no other significant correlations were found in the two SZ patient groups. Detailed results are given in online Supplementary Tables 13 and 14.

## Discussion

The preliminary efficacy of Komori Life, a new online training program, was tested among adults with schizophrenia. Following 60 min of training performed over a 4-week period, our results showed that, relative to the TAU group, the GT group (Komori Life training) revealed greater improvement in eye movement measures, including the percentage of total duration and percentage of total fixations towards threatening stimuli, suggesting ameliorating effects on the severity of cognitive impairments in patients with schizophrenia assigned to the GT group. However, no significant changes were observed in cognitive or clinical assessments.

These findings are in agreement with those of previous studies on therapeutic game interventions and suggest that an online training program is feasible and beneficial for schizophrenia [16, 24]. Relative to traditional cognitive interventions that were conducted in group settings in the clinic, supervised by well-trained clinicians [45], online game interventions can be applied remotely, such as in the patient’s home. In this way, patients could reduce wait times for medical appointments and continuously receive a therapeutic intervention when face-to face interaction with a therapist is not possible, especially during the COVID-19 pandemic [24]. Additionally, interesting game interventions may enhance motivation and adherence, which adds weight to the idea that this individualized online training program is a feasible way to target cognitive impairment among patients with schizophrenia [46]. This intervention can be combined with other treatments offering additional benefits in standard care. Of note, the present results are preliminary, and more large, well-controlled trials are required to confirm whether the therapeutic effects of applying Komori Life are replicable.

The results of our intervention for cognitive impairment were mixed. On the one hand, regarding the cognitive and clinical assessments, there were no significant differences in cognitive or clinical assessments at baseline between the two SZ patient groups, indicating that the two groups had the same level of cognitive impairments before the intervention. However, there were no significant group interactions in improvements among cognitive and clinical scores after game training. These results are in agreement with negative effects found for a range of cognitive measures in relatively large, well-controlled trials of computerized cognitive intervention for schizophrenia [25, 47]. Possible reasons for the null effects are considered below. First, due to the relatively short study duration, our training program only lasted for 60 min each time over 4 weeks, and most previous studies that have reported positive effects on cognitive function had longer training durations varying from 8 weeks to 12 weeks [26]. This study duration may be too short to raise a detectable change for cognitive and clinical assessments. Second, incompleteness of outcome data, due to several reasons, including poor understanding and discharge from the hospital before posttraining assessments, cognitive and clinical outcome data failed to be collected from some patients, and the missing values were higher than 15%, a level that would make the findings statistically questionable [25]. Additionally, this could be accounted for by the nature of the assessment itself, which may not be sensitive enough to detect cognitive changes arising from training, or due to outdated tasks relative to the amazing technological advances in the modern era [16]. Additional advanced evaluation methods may be required to accurately capture changes for cognitive improvement.

On the other hand, only the GT group improved in eye movement measurements. At baseline, there were no significant differences in eye movement measurements between the two SZ patient groups, suggesting that the two groups were well randomized in terms of baseline characteristics. Consistent with prior studies, both patient groups showed a higher percentage of total fixations and total duration towards threatening scenes compared to controls, indicating an attentional bias towards threatening scenes in schizophrenia [32]. Moreover, two patient groups also showed higher gaze duration on threatening scenes [33, 34]. According to previous theories, the percentage of total fixations and total duration reflect attentional maintenance. Gaze duration reflect attentional engagement. These findings are congruent with previous studies, demonstrating an attentional engagement and maintenance bias toward threat-related stimuli [33, 34]. Heightened attentional maintenance to threat has been shown to be associated with the severity of schizophrenia [48]. The severity of schizophrenia was associated with high-order cognitive impairments, social cognition impairments, and affective deficits that may result in desensitization to threatening stimuli, more time evaluating threatening information, and difficulty avoiding threatening cues once it has been captured by a negative stimulus [32]. All these factors could result in attentional biases towards threatening stimuli. In sum, attentional biases to threat showed a strong link with cognitive impairments in schizophrenia. Therefore, it might be an objective marker for evaluating treatment outcomes in schizophrenia.

Following our hypothesis, only the GT group showed a decreasing trend for attentional biases towards threatening stimuli and an increasing trend for attentional maintenance to happy stimuli at the posttraining assessment. After controlling for potential confounding variables such as age, sex, and education level, these results still existed, suggesting an attentional maintenance bias away from negative information in the GT group. Therefore, relative to the TAU group, atypical attentional patterns to emotional information were ameliorated in the GT group. These results suggest that game intervention may improve cognitive function [24] and that this improvement may lead to sensitization to threatening stimuli and a lower propensity to persevere in negative information [49]. Moreover, training effectiveness was associated with cognitive improvement, and heightened attentional maintenance to threat was associated with worse cognitive performance, although these correlations did not survive multiple corrections. These associations further indicated that game training could improve cognitive function in people with schizophrenia but should be interpreted with caution due to nonsignificant results after multiple comparisons.

Some limitations of this study need to be mentioned. First, our training program was completed in a relatively short period of time, which may result in negative results for cognitive and clinical assessments. It is possible that positive effects will be found in a longer period of training. Furthermore, we cannot rule out the possibility that improvements are driven by nonspecific effects of training, the so-called Hawthorne effect [50]. These effects may limit our ability to confirm the effect of game training on cognitive improvements. An active control game may be desirable in future trials of CRT to control nonspecific factors such as special attention. In any case, any confounding effects this might have produced were compensated for by the eye movement results. We are reasonably confident about the observed significant effects. Finally, this study lacked follow-up and did not combine game training with other cognitive interventions. Future studies should consider a comprehensive training program including multicomponent cognitive interventions that might be more efficacious than a single training strategy.

In conclusion, the current study provides evidence that a promising online intervention is feasible and may be efficacious in improving both cognitive performance and everyday functioning among people diagnosed with schizophrenia. Given that effective interventions remain limited, this intervention may serve as a complementary therapy to standard psychiatric care. Nevertheless, future randomized controlled trials are needed to determine whether these preliminary findings are replicable.

### Supplementary information

SUPPLEMENTAL MATERIAL

## Data Availability

The data that support the findings of this study are available from corresponding author upon reasonable request.
