# Supplementary material for: Is game-based therapy effective for treating cognitive deficits in adults with schizophrenia? Evidence from a randomized controlled trial
Source: Transl Psychiatry. 2024 Jul 16;14:291. doi: 10.1038/s41398-024-02920-0 (PMC11252351; doi:10.1038/s41398-024-02920-0)
Supplement: Supplementary file 1 — SUPPLEMENTAL MATERIAL [file 41398_2024_2920_MOESM1_ESM.docx]

**Is game-based therapy effective for treating cognitive deficits in adults with schizophrenia? Evidence from a randomized controlled trial**

**Supplemental Data:**

Table S1 Cognitive and clinical assessments at baseline between two SZ groups

|  | GT | TAU | *P* Value |
| --- | --- | --- | --- |
| PANSS, mean (SD) | 55.13 (16.03) | 57.31 (14.34) | 0.693 |
| BNSS, mean (SD) | 22.52 (15.65) | 25.05 (13.01) | 0.450 |
| PSP, mean (SD) | 65.21 (11.81) | 62.43 (13.13) | 0.338 |
| TEPS, mean (SD) | 77.44 (13.34) | 77.56 (16.24) | 0.972 |
| SES, mean (SD) | 28.19 (2.67) | 27.74 (2.80) | 0.491 |
| SQLS, mean (SD) | 21.65 (11.75) | 26.09 (14.35) | 0.159 |
| MCCB, mean (SD) | 41.28 (8.82) | 39.42 (8.18) | 0.426 |

Note: The two-sample t-test was applied to test for group differences, statistical significance level was set at *P* < 0.05 (two-tailed). Abbreviations: GT, game training; TAU, treatment as Usual; PANSS, positive and negative syndrome scale; BNSS, brief negative symptoms scale; PSP, personal and social performance scale; TEPS, temporal experience of pleasure scale; SES, self-esteem scale; SQLS, schizophrenia quality of life scale; MCCB, measurement and treatment research to improve cognition schizophrenia (MATRICS) consensus cognitive battery.

Table S2 Cognitive and clinical assessments after training between two SZ groups

|  | GT | TAU | F | df | *P* | η² |
| --- | --- | --- | --- | --- | --- | --- |
| PANSS, mean (SD) | 49.56 (10.10) | 48.48 (10.22) |  |  |  |  |
| Group |  |  | 0.663 | 1, 25 | 0.423 | 0.026 |
| Time |  |  | 3.436 | 1, 25 | 0.076 | 0.121 |
| Time × Group |  |  | 0.062 | 1, 25 | 0.806 | 0.002 |
| BNSS, mean (SD) | 19.11 (13.16) | 20.33 (10.73) |  |  |  |  |
| Group |  |  | 0.133 | 1, 25 | 0.718 | 0.005 |
| Time |  |  | 2.016 | 1, 25 | 0.168 | 0.075 |
| Time × Group |  |  | 0.370 | 1, 25 | 0.548 | 0.015 |
| PSP, mean (SD) | 71.36 (8.29) | 69.21 (9.12) |  |  |  |  |
| Group |  |  | 0.002 | 1, 26 | 0.966 | 0.000 |
| Time |  |  | **10.598** | **1, 26** | **0.003** | **0.290** |
| Time × Group |  |  | 2.308 | 1, 26 | 0.141 | 0.082 |
| TEPS, mean (SD) | 76.74 (17.88) | 77.03 (15.84) |  |  |  |  |
| Group |  |  | 0.012 | 1, 29 | 0.915 | 0.000 |
| Time |  |  | 0.012 | 1, 29 | 0.913 | 0.000 |
| Time × Group |  |  | 0.027 | 1, 29 | 0.871 | 0.001 |
| SES, mean (SD) | 28.27 (2.66) | 28.31 (3.74) |  |  |  |  |
| Group |  |  | 0.069 | 1, 27 | 0.795 | 0.003 |
| Time |  |  | 0.002 | 1, 27 | 0.965 | 0.000 |
| Time × Group |  |  | 0.340 | 1, 27 | 0.565 | 0.012 |
| SQLS, mean (SD) | 21.00 (12.02) | 22.23 (12.55) |  |  |  |  |
| Group |  |  | 1.935 | 1, 28 | 0.175 | 0.065 |
| Time |  |  | 0.617 | 1, 28 | 0.439 | 0.022 |
| Time × Group |  |  | 0.130 | 1, 28 | 0.721 | 0.005 |
| MCCB, mean (SD) | 39.56 (12.45) | 39.83 (11.96) |  |  |  |  |
| Group |  |  | 1.286 | 1, 21 | 0.270 | 0.058 |
| Time |  |  | 0.126 | 1, 21 | 0.726 | 0.006 |
| Time × Group |  |  | 0.126 | 1, 21 | 0.726 | 0.006 |

Note: The two-way repeated-measures ANOVA was applied to test for group differences, statistical significance level was set at *P* < 0.05 (two-tailed). Significant *P*-values were in bold. Abbreviations: GT, game training; TAU, treatment as Usual; PANSS, positive and negative syndrome scale; BNSS, brief negative symptoms scale; PSP, personal and social performance scale; TEPS, temporal experience of pleasure scale; SES, self-esteem scale; SQLS, schizophrenia quality of life scale; MCCB, measurement and treatment research to improve cognition schizophrenia (MATRICS) consensus cognitive battery.

Table S3 Mean (standard deviation) for the percentage of total duration, and percentage of total fixations for sad, happy, threatening, or neutral conditions in the three groups

| Conditions | GT | TAU | HC |
| --- | --- | --- | --- |
| Baseline |  |  |  |
| The percentage of total duration |  |  |  |
| Happy condition | 25.44 (6.59) | 23.79 (7.25) | 29.48 (10.36) |
| Threatening condition | 29.37 (5.71) | 28.39 (7.12) | 21.12 (8.50) |
| Sad condition | 28.37（1.71 | 32.31（1.67 | 28.51（1.50 |
| Neutral condition | 16.82（1.66 | 15.51（1.62 | 20.89（1.56 |
| The percentage of total fixations |  |  |  |
| Happy condition | 24.55 (4.83) | 22.45 (4.56) | 26.74 (6.87) |
| Threatening condition | 29.76 (4.90) | 29.20 (4.85) | 22.00 (7.59) |
| Sad condition | 27.37 (1.09) | 28.60 (1.07) | 29.81 (1.10) |
| Neutral condition | 18.31 (1.36) | 19.75 (1.33) | 21.45 (1.40) |
| After training |  |  |  |
| The percentage of total duration |  |  |  |
| Happy condition | 30.74 (5.38) | 24.05 (5.17) |  |
| Threatening condition | 25.69 (4.30) | 32.11 (8.45) |  |
| Sad condition | 27.67 (1.59) | 28.35 (1.56) |  |
| Neutral condition | 15.90 (1.57) | 15.49 (1.53) |  |
| The percentage of total fixations |  |  |  |
| Happy condition | 27.54 (4.67) | 22.80 (5.26) |  |
| Threatening condition | 26.70 (4.16) | 32.78 (7.17) |  |
| Sad condition | 28.24 (1.47) | 27.53 (1.43) |  |
| Neutral condition | 17.53 (1.44) | 16.89 (1.41) |  |

Note: Abbreviations: GT, game training; TAU, treatment as Usual; HC, healthy controls.

Table S4 Mean (standard deviation) for the first-pass fixations, percentage of first fixation and gaze duration in the three groups at baseline

| Conditions | GT | TAU | HC |
| --- | --- | --- | --- |
| **The percentage of first fixation** |  |  |  |
| Happy condition | 23.77 (4.25) | 21.33 (3.31) | 23.75 (5.98) |
| Threatening condition | 26.91 (5.20) | 30.40 (4.44) | 29.36 (5.48) |
| Sad condition | 28.06 (4.80) | 28.01 (5.31) | 22.98 (8.51) |
| Neutral condition | 21.27 (5.33) | 20.26 (4.57) | 23.91 (7.36) |
| **The first-pass fixations** |  |  |  |
| Happy condition | 1.41 (0.35) | 1.33 (0.50) | 1.65 (0.70) |
| Threatening condition | 1.66 (0.59) | 1.96 (0.83) | 2.07 (0.81) |
| Sad condition | 1.75 (0.67) | 1.79 (0.80) | 1.66 (0.93) |
| Neutral condition | 1.26 (0.40) | 1.22 (0.41) | 1.64 (0.69) |
| **Gaze duration** |  |  |  |
| Happy condition | 0.77 (0.44) | 0.83 (0.46) | 0.42 (0.22) |
| Threatening condition | 0.83 (0.57) | 0.84 (0.32) | 0.36 (0.17) |
| Sad condition | 0.87 (0.42) | 0.81 (0.34) | 0.30 (0.21) |
| Neutral condition | 0.80 (0.43) | 0.61 (0.39) | 0.29 (0.17) |

Note: Abbreviations: GT, game training; TAU, treatment as Usual; HC, healthy controls.

Table S5 All main effects and interactions of ANOVAs in the three groups at baseline

| Group | Eye-tracker parameter effect | F | df | *P* | η² |
| --- | --- | --- | --- | --- | --- |
| TAU vs GT vs HC | **The percentage of total duration** |  |  |  |  |
|  | Group | 0.000 | 2, 252 | 1. 000 | 0. 000 |
|  | Valence | **27.233** | **3, 252** | **0. 000** | **0. 245** |
|  | Group x Valence | **4.942** | **6, 252** | **0. 000** | **0. 105** |
|  | **The percentage of total fixations** |  |  |  |  |
|  | Group | 0.000 | 2, 252 | 1. 000 | 0. 000 |
|  | Valence | **27.123** | **3, 252** | **0.000** | **0.244** |
|  | Group x Valence | **5. 744** | **6, 252** | **0. 000** | **0. 120** |
|  | **The percentage of first fixation** |  |  |  |  |
|  | Group | 0.000 | 2, 252 | 1. 000 | 0. 000 |
|  | Valence | **3.304** | **3, 252** | **0.029** | **0.075** |
|  | Group x Valence | 1.843 | 6, 252 | 0.151 | 0.044 |
|  | **The first-pass fixations** |  |  |  |  |
|  | Group | 2.862 | 2, 252 | 0.059 | 0.022 |
|  | Valence | **8.099** | **3, 252** | **0.000** | **0.088** |
|  | Group x Valence | 1.119 | 6, 252 | 0.352 | 0.026 |
|  | **Gaze duration** |  |  |  |  |
|  | Group | **44.217** | **2, 252** | **0.000** | **0.260** |
|  | Valence | 1.310 | 3, 252 | 0.272 | 0.015 |
|  | Group x Valence | 0.718 | 6, 252 | 0.636 | 0.017 |

Note: The two-way ANOVA was applied to test for group differences, statistical significance level was set at *P* < 0.05 (two-tailed). Significant *P*-values were in bold. Abbreviations: GT, game training; TAU, treatment as Usual; HC, healthy controls.

Table S6 All main effects and interactions of ANOVAs in the three groups at baseline after controlling for age, sex and education level

| Group | Eye-tracker parameter effect | F | df | *P* | η² |
| --- | --- | --- | --- | --- | --- |
| TAU vs GT vs HC | **The percentage of total duration** |  |  |  |  |
|  | Group | 0.000 | 2, 249 | 1. 000 | 0. 000 |
|  | Valence | **26.909** | **3,** 249 | **0. 000** | **0. 245** |
|  | Group x Valence | **4.883** | **6,** 249 | **0. 000** | **0. 105** |
|  | **The percentage of total fixations** |  |  |  |  |
|  | Group | 0.000 | 2, 249 | 1. 000 | 0. 000 |
|  | Valence | **26.800** | **3,** 249 | **0.000** | **0.244** |
|  | Group x Valence | **5.675** | **6,** 249 | **0. 000** | **0. 120** |
|  | **The percentage of first fixation** |  |  |  |  |
|  | Group | 0.000 | 2, 249 | 1. 000 | 0. 000 |
|  | Valence | 2.167 | **3,** 249 | 0102 | 0.053 |
|  | Group x Valence | 1.633 | **6,** 249 | 0.190 | 0.040 |
|  | **The first-pass fixations** |  |  |  |  |
|  | Group | 7.942 | 2, 249 | 0.000 | 0.060 |
|  | Valence | 8.648 | **3,** 249 | 0.000 | 0.094 |
|  | Group x Valence | 1.195 | **6,** 249 | 0.310 | 0.028 |
|  | **Gaze duration** |  |  |  |  |
|  | Group | 42.578 | 2, 249 | 0.000 | 0.255 |
|  | Valence | 1.330 | **3,** 249 | 0.265 | 0.016 |
|  | Group x Valence | 0.729 | **6,** 249 | 0.627 | 0.017 |

Note: The ANCOVA was applied to test for group differences, statistical significance level was set at *P* < 0.05 (two-tailed). Significant *P*-values were in bold. Abbreviations: GT, game training; TAU, treatment as Usual; HC, healthy controls.

Table S7 The main effects and interactions of ANOVAs for threatening and happy conditions in the two SZ patient groups after training

| Eye-tracker parameter effect | F | df | *P* | η² |
| --- | --- | --- | --- | --- |
| Happy condition |  |  |  |  |
| **The percentage of total duration** |  |  |  |  |
| Group | **7.282** | **1, 41** | **0.010** | **0.151** |
| Time | **5.872** | **1, 41** | **0.020** | **0.125** |
| Group x Time | **4.812** | **1, 41** | **0.034** | **0.105** |
| **The percentage of total fixations** |  |  |  |  |
| Group | **9.062** | **1, 41** | **0.004** | **0.181** |
| Time | 2.793 | 1, 41 | 0.102 | 0.064 |
| Group x Time | 1.742 | 1, 41 | 0.194 | 0.041 |
| Threatening condition |  |  |  |  |
| **The percentage of total duration** |  |  |  |  |
| Group | 3.340 | 1, 41 | 0.075 | 0.075 |
| Time | 0.000 | 1, 41 | 0.992 | 0.000 |
| Group x Time | **6.688** | **1, 41** | **0.013** | **0.140** |
| **The percentage of total fixations** |  |  |  |  |
| Group | **6.046** | **1, 41** | **0.018** | **0.129** |
| Time | 0.042 | 1, 41 | 0.839 | 0.001 |
| Group x Time | **6.945** | **1, 41** | **0.012** | **0.145** |

Note: The two-way repeated-measures ANOVA was applied to test for group differences, statistical significance level was set at *P* < 0.05 (two-tailed). Significant *P*-values were in bold.

Table S8 The main effects and interactions of ANOVAs for threatening and happy conditions in the two SZ patient groups after training controlling for age, sex and education level

| Eye-tracker parameter effect | F | df | *P* | η² |
| --- | --- | --- | --- | --- |
| Happy condition |  |  |  |  |
| **The percentage of total duration** |  |  |  |  |
| Group | **5.900** | **1, 39** | **0.020** | **0.131** |
| Time | 0.008 | 1, 39 | 0.930 | 0.000 |
| Group x Time | **5.421** | **1, 39** | **0.025** | **0.122** |
| **The percentage of total fixations** |  |  |  |  |
| Group | **9.564** | **1, 39** | **0.004** | **0.197** |
| Time | 2.410 | 1, 39 | 0.129 | 0.058 |
| Group x Time | 1.818 | 1, 39 | 0.185 | 0.045 |
| Threatening condition |  |  |  |  |
| **The percentage of total duration** |  |  |  |  |
| Group | 1.963 | 1, 39 | 0.169 | 0.048 |
| Time | 0.156 | 1, 39 | 0.695 | 0.004 |
| Group x Time | **6.701** | **1, 39** | **0.013** | **0.147** |
| **The percentage of total fixations** |  |  |  |  |
| Group | **4.476** | **1, 39** | **0.041** | **0.103** |
| Time | 0.109 | 1, 39 | 0.743 | 0.003 |
| Group x Time | **6.104** | **1, 39** | **0.018** | **0.135** |

Note: The two-way repeated-measures ANOVA was applied to test for group differences, statistical significance level was set at *P* < 0.05 (two-tailed). Significant *P*-values were in bold.

Table S9 Mean (standard deviation) for the first-pass fixations, percentage of first fixation and gaze duration in the two SZ patient after training

| Conditions | GT | TAU |
| --- | --- | --- |
| **The percentage of first fixation** |  |  |
| Happy condition | 24.73 (5.07) | 23.35 (5.17) |
| Threatening condition | 29.43 (6.11) | 29.11 (8.05) |
| Sad condition | 27.68 (4.24) | 28.72 (5.89) |
| Neutral condition | 18.16 (7.05) | 18.82 (7.20) |
| **The first-pass fixations** |  |  |
| Happy condition | 2.32 (1.04) | 2.13 (0.68) |
| Threatening condition | 2.84 (1.36) | 2.91 (1.68) |
| Sad condition | 2.65 (1.23) | 2.80 (1.34) |
| Neutral condition | 1.57 (0.64) | 1.63 (0.59) |
| **Gaze duration** |  |  |
| Happy condition | 0.41 (0.23) | 0.47 (0.33) |
| Threatening condition | 0.37 (0.13) | 0.40 (0.17) |
| Sad condition | 0.29 (0.11) | 0.31 (0.10) |
| Neutral condition | 0.31 (0.17) | 0.32 (0.16) |

Note: Abbreviations: GT, game training; TAU, treatment as Usual.

Table S10 The main effects and interactions of ANOVAs for sad and neutral conditions in the two SZ patient groups after training

| Eye-tracker parameter effect | F | df | *P* | η² |
| --- | --- | --- | --- | --- |
| Sad condition |  |  |  |  |
| **The percentage of total duration** |  |  |  |  |
| Group | 1.401 | 1, 39 | 0.244 | 0.035 |
| Time | 0.374 | 3, 39 | 0.544 | 0.010 |
| Group x Time | 1.546 | 3, 39 | 0.221 | 0.038 |
| **The percentage of total fixations** |  |  |  |  |
| Group | 0.032 | 1, 39 | 0.858 | 0.001 |
| Time | 1.293 | 3, 39 | 0.262 | 0.032 |
| Group x Time | 0.779 | 3, 39 | 0.383 | 0.020 |
| Neutral condition |  |  |  |  |
| **The percentage of total duration** |  |  |  |  |
| Group | 0.082 | 1, 39 | 0.776 | 0.002 |
| Time | 1.303 | 3, 39 | 0.261 | 0.032 |
| Group x Time | 0.157 | 3, 39 | 0.694 | 0.004 |
| **The percentage of total fixations** |  |  |  |  |
| Group | 0.261 | 1, 39 | 0.612 | 0.007 |
| Time | **4.491** | **3, 39** | **0.040** | **0.103** |
| Group x Time | 0.696 | 3, 39 | 0.409 | 0.018 |

Note: The two-way repeated-measures ANOVA was applied to test for group differences, statistical significance level was set at *P* < 0.05 (two-tailed). Significant *P*-values were in bold.

Table S11 All main effects and interactions of ANOVAs for the first-pass fixations, percentage of first fixation and gaze duration in the two SZ patient groups after training

| Conditions | Eye-tracker parameter effect | F | df | *P* | η² |
| --- | --- | --- | --- | --- | --- |
| Happy condition | **The percentage of first fixation** |  |  |  |  |
|  | Group | 2. 471 | 1, 39 | 0. 124 | 0. 060 |
|  | Time | 0. 000 | 3, 39 | 0. 994 | 0. 000 |
|  | Group x Time | 0. 174 | 3, 39 | 0. 679 | 0. 004 |
|  | **The first-pass fixations** |  |  |  |  |
|  | Group | 1. 921 | 1, 39 | 0. 174 | 0. 047 |
|  | Time | 2. 290 | 3, 39 | 0. 138 | 0. 055 |
|  | Group x Time | 0. 189 | 3, 39 | 0. 666 | 0. 005 |
|  | **Gaze duration** |  |  |  |  |
|  | Group | 0. 711 | 1, 39 | 0. 404 | 0. 018 |
|  | Time | 1. 957 | 3, 39 | 0. 170 | 0. 048 |
|  | Group x Time | 0. 026 | 3, 39 | 0. 872 | 0. 001 |
| Threatening condition | **The percentage of first fixation** |  |  |  |  |
|  | Group | 0. 011 | 1, 39 | 0. 917 | 0. 000 |
|  | Time | 1. 761 | 3, 39 | 0. 192 | 0. 043 |
|  | Group x Time | 0. 139 | 3, 39 | 0. 711 | 0. 004 |
|  | **The first-pass fixations** |  |  |  |  |
|  | Group | 0. 045 | 1, 39 | 0. 833 | 0. 001 |
|  | Time | 3. 762 | 3, 39 | 0. 060 | 0. 088 |
|  | Group x Time | 0. 019 | 3, 39 | 0. 890 | 0. 000 |
|  | **Gaze duration** |  |  |  |  |
|  | Group | 0. 151 | 1, 39 | 0. 700 | 0. 004 |
|  | Time | **9. 106** | 3, 39 | **0. 004** | **0. 189** |
|  | Group x Time | 0. 662 | 3, 39 | 0. 421 | 0. 017 |
| Sad condition | **The percentage of first fixation** |  |  |  |  |
|  | Group | 0. 673 | 1, 39 | 0. 417 | 0. 017 |
|  | Time | 0. 038 | 3, 39 | 0. 847 | 0. 001 |
|  | Group x Time | 2. 210 | 3, 39 | 0. 145 | 0. 054 |
|  | **The first-pass fixations** |  |  |  |  |
|  | Group | 0. 017 | 1, 39 | 0. 896 | 0. 000 |
|  | Time | 2. 676 | 3, 39 | 0. 110 | 0. 064 |
|  | Group x Time | 0. 258 | 3, 39 | 0. 614 | 0. 007 |
|  | **Gaze duration** |  |  |  |  |
|  | Group | 0. 281 | 1, 39 | 0. 599 | 0. 007 |
|  | Time | 0. 105 | 3, 39 | 0. 748 | 0. 003 |
|  | Group x Time | 0. 001 | 3, 39 | 0. 981 | 0. 000 |
| Neutral condition | **The percentage of first fixation** |  |  |  |  |
|  | Group | 0. 048 | 1, 39 | 0. 827 | 0. 001 |
|  | Time | 1. 872 | 3, 39 | 0. 179 | 0. 046 |
|  | Group x Time | 0. 534 | 3, 39 | 0. 469 | 0. 014 |
|  | **The first-pass fixations** |  |  |  |  |
|  | Group | 0. 039 | 1, 39 | 0. 844 | 0. 001 |
|  | Time | 0. 005 | 3, 39 | 0. 946 | 0. 000 |
|  | Group x Time | 0. 416 | 3, 39 | 0. 523 | 0. 011 |
|  | **Gaze duration** |  |  |  |  |
|  | Group | 0. 751 | 1, 39 | 0. 391 | 0. 019 |
|  | Time | 0. 269 | 3, 39 | 0. 607 | 0. 007 |
|  | Group x Time | 1. 198 | 3, 39 | 0. 281 | 0. 030 |

Note: The two-way repeated-measures ANOVA was applied to test for group differences, statistical significance level was set at *P* < 0.05 (two-tailed). Significant *P*-values were in bold.

Table S12 Cognitive assessments after training between two SZ groups

| Cognitive tests | F | df | *P* | η² |
| --- | --- | --- | --- | --- |
| **Trail making test** |  |  |  |  |
| Group | 0.715 | 1, 21 | 0.407 | 0.033 |
| Time | 0.297 | 1, 21 | 0.591 | 0.014 |
| Time × Group | 1.032 | 1, 21 | 0.321 | 0.047 |
| **Symbol coding** |  |  |  |  |
| Group | 0.358 | 1, 21 | 0.557 | 0.019 |
| Time | 0.296 | 1, 21 | 0.593 | 0.015 |
| Time × Group | 0.117 | 1, 21 | 0.736 | 0.006 |
| **Verbal learning test** |  |  |  |  |
| Group | 0.572 | 1, 21 | 0.459 | 0.029 |
| Time | 0.351 | 1, 21 | 0.561 | 0.018 |
| Time × Group | 1.177 | 1, 21 | 0.291 | 0.058 |
| **Spatial span** |  |  |  |  |
| Group | 0.725 | 1, 21 | 0.405 | 0.037 |
| Time | 0.534 | 1, 21 | 0.474 | 0.027 |
| Time × Group | 4.007 | 1, 21 | 0.060 | 0.174 |
| **Identical pairs** |  |  |  |  |
| Group | 1.061 | 1, 21 | 0.316 | 0.053 |
| Time | 3.278 | 1, 21 | 0.086 | 0.147 |
| Time × Group | 3.678 | 1, 21 | 0.070 | 0.162 |
| **Mazes** |  |  |  |  |
| Group | 0.428 | 1, 21 | 0.521 | 0.022 |
| Time | 0.688 | 1, 21 | 0.417 | 0.035 |
| Time × Group | 0.587 | 1, 21 | 0.453 | 0.030 |
| **Visuospatial memory test** |  |  |  |  |
| Group | 1.433 | 1, 21 | 0.246 | 0.070 |
| Time | 2.134 | 1, 21 | 0.160 | 0.101 |
| Time × Group | 0.338 | 1, 21 | 0.568 | 0.017 |
| **Animal naming (Fluency)** |  |  |  |  |
| Group | 0.362 | 1, 21 | 0.554 | 0.019 |
| Time | 2.475 | 1, 21 | 0.132 | 0.115 |
| Time × Group | 0.060 | 1, 21 | 0.809 | 0.003 |
| **Managing emotions** |  |  |  |  |
| Group | 0.334 | 1, 21 | 0.570 | 0.017 |
| Time | 0.421 | 1, 21 | 0.524 | 0.022 |
| Time × Group | 0.000 | 1, 21 | 0.983 | 0.000 |

Note: The two-way repeated-measures ANOVA was applied to test for group differences, statistical significance level was set at *P* < 0.05 (two-tailed). Significant *P*-values were in bold.

Table S13 Correlation results among game-related measures, cognitive and clinical improvement scores, and eye movement improvement scores in the two SZ patient groups

| Group | Variable 1 | Variable 2 | Correlation Coefficient | *P* value |
| --- | --- | --- | --- | --- |
| GT | game-related measures | eye movement improvement scores |  |  |
|  | Game grades | The percentage of total duration on threatening stimuli changes | -0.125 | 0.657 |
|  | Total playing time | The percentage of total duration on threatening stimuli changes | 0.304 | 0.220 |
|  | Total game playing behaviors | The percentage of total duration on threatening stimuli changes | 0.441 | 0.067 |
|  | Active degree | The percentage of total duration on threatening stimuli changes | -0.201 | 0.472 |
|  | Game grades | The percentage of total fixations on threatening stimuli changes | -0.273 | 0.325 |
|  | Total playing time | The percentage of total fixations on threatening stimuli changes | 0.204 | 0.417 |
|  | Total game playing behaviors | The percentage of total fixations on threatening stimuli changes | 0.291 | 0.241 |
|  | Active degree | The percentage of total fixations on threatening stimuli changes | -0.272 | 0.326 |
|  | Game grades | The percentage of total duration on happy stimuli changes | 0.297 | 0.282 |
|  | Total playing time | The percentage of total duration on happy stimuli changes | -0.076 | 0.763 |
|  | Total game playing behaviors | The percentage of total duration on happy stimuli changes | -0.041 | 0.873 |
|  | Active degree | The percentage of total duration on happy stimuli changes | -0.079 | 0.780 |
|  | Game grades | The percentage of total fixations on happy stimuli changes | 0.461 | 0.084 |
|  | Total playing time | The percentage of total fixations on happy stimuli changes | 0.280 | 0.261 |
|  | Total game playing behaviors | The percentage of total fixations on happy stimuli changes | 0.372 | 0.128 |
|  | Active degree | The percentage of total fixations on happy stimuli changes | 0.239 | 0.391 |
|  | PANSS | The percentage of total duration on threatening stimuli changes | 0. 171 | 0. 511 |
|  | BNSS | The percentage of total duration on threatening stimuli changes | -0. 263 | 0. 307 |
|  | PSP | The percentage of total duration on threatening stimuli changes | -0. 136 | 0. 629 |
|  | TEPS | The percentage of total duration on threatening stimuli changes | -0. 325 | 0. 220 |
|  | SES | The percentage of total duration on threatening stimuli changes | -0. 347 | 0. 172 |
|  | SQLS | The percentage of total duration on threatening stimuli changes | 0. 260 | 0. 332 |
|  | MCCB | The percentage of total duration on threatening stimuli changes | -0. 094 | 0. 772 |
|  | PANSS | The percentage of total fixations on threatening stimuli changes | -0. 011 | 0. 966 |
|  | PSP | The percentage of total fixations on threatening stimuli changes | -0. 208 | 0. 456 |
|  | TEPS | The percentage of total fixations on threatening stimuli changes | -0. 040 | 0. 882 |
|  | SES | The percentage of total fixations on threatening stimuli changes | -0. 132 | 0. 612 |
|  | SQLS | The percentage of total fixations on threatening stimuli changes | -0. 266 | 0. 319 |
|  | MCCB | The percentage of total fixations on threatening stimuli changes | -0. 020 | 0. 952 |
|  | PANSS | The percentage of total duration on happy stimuli changes | 0. 130 | 0. 618 |
|  | BNSS | The percentage of total duration on happy stimuli changes | -0. 079 | 0. 762 |
|  | PSP | The percentage of total duration on happy stimuli changes | -0. 174 | 0. 536 |
|  | TEPS | The percentage of total duration on happy stimuli changes | 0. 218 | 0. 418 |
|  | SES | The percentage of total duration on happy stimuli changes | 0. 259 | 0. 315 |
|  | SQLS | The percentage of total duration on happy stimuli changes | -0. 053 | 0. 845 |
|  | MCCB | The percentage of total duration on happy stimuli changes | 0. 385 | 0. 216 |
|  | BNSS | The percentage of total fixations on happy stimuli changes | 0. 213 | 0. 412 |
|  | PSP | The percentage of total fixations on happy stimuli changes | -0. 023 | 0. 935 |
|  | TEPS | The percentage of total fixations on happy stimuli changes | -0. 249 | 0. 352 |
|  | SES | The percentage of total fixations on happy stimuli changes | -0. 225 | 0. 386 |
|  | SQLS | The percentage of total fixations on happy stimuli changes | 0. 308 | 0. 246 |
|  | MCCB | The percentage of total fixations on happy stimuli changes | 0. 252 | 0. 430 |
| TAU |  |  |  |  |
|  | PANSS | The percentage of total duration on threatening stimuli changes | 0.015 | 0.959 |
|  | BNSS | The percentage of total duration on threatening stimuli changes | 0.173 | 0.538 |
|  | PSP | The percentage of total duration on threatening stimuli changes | -0.228 | 0.434 |
|  | TEPS | The percentage of total duration on threatening stimuli changes | 0.182 | 0.515 |
|  | SES | The percentage of total duration on threatening stimuli changes | 0.385 | 0.174 |
|  | SQLS | The percentage of total duration on threatening stimuli changes | 0.159 | 0.570 |
|  | MCCB | The percentage of total duration on threatening stimuli changes | 0.600 | 0.154 |
|  | PANSS | The percentage of total fixations on threatening stimuli changes | -0.366 | 0.180 |
|  | BNSS | The percentage of total fixations on threatening stimuli changes | 0.191 | 0.496 |
|  | PSP | The percentage of total fixations on threatening stimuli changes | 0.127 | 0.664 |
|  | TEPS | The percentage of total fixations on threatening stimuli changes | 0.324 | 0.239 |
|  | SES | The percentage of total fixations on threatening stimuli changes | 0.525 | 0.054 |
|  | SQLS | The percentage of total fixations on threatening stimuli changes | -0.120 | 0.669 |
|  | MCCB | The percentage of total fixations on threatening stimuli changes | -0.050 | 0.915 |
|  | PANSS | The percentage of total duration on happy stimuli changes | -0.313 | 0.255 |
|  | BNSS | The percentage of total duration on happy stimuli changes | -0.032 | 0.911 |
|  | PSP | The percentage of total duration on happy stimuli changes | 0.267 | 0.356 |
|  | TEPS | The percentage of total duration on happy stimuli changes | 0.001 | 0.997 |
|  | SES | The percentage of total duration on happy stimuli changes | 0.261 | 0.367 |
|  | SQLS | The percentage of total duration on happy stimuli changes | -0.600 | 0.018 |
|  | MCCB | The percentage of total duration on happy stimuli changes | -0.444 | 0.318 |
|  | PANSS | The percentage of total fixations on happy stimuli changes | 0.197 | 0.482 |
|  | BNSS | The percentage of total fixations on happy stimuli changes | -0.178 | 0.525 |
|  | PSP | The percentage of total fixations on happy stimuli changes | -0.340 | 0.235 |
|  | TEPS | The percentage of total fixations on happy stimuli changes | -0.308 | 0.264 |
|  | SES | The percentage of total fixations on happy stimuli changes | -0.143 | 0.625 |
|  | SQLS | The percentage of total fixations on happy stimuli changes | 0.148 | 0.600 |
|  | MCCB | The percentage of total fixations on happy stimuli changes | 0.462 | 0.297 |

Note: Abbreviations: GT, game training; TAU, treatment as usual; PANSS, positive and negative syndrome scale; BNSS, brief negative symptoms scale; PSP, personal and social performance scale; TEPS, temporal experience of pleasure scale; SES, self-esteem scale; SQLS, schizophrenia quality of life scale; MCCB, measurement and treatment research to improve cognition schizophrenia (MATRICS) consensus cognitive battery.

Table S14 Partial correlation results among game-related measures, cognitive and clinical improvement scores, and eye movement improvement scores in the two SZ patient groups after controlling for age, sex and education level

| Group | Variable 1 | Variable 2 | Correlation Coefficient | *P* value |
| --- | --- | --- | --- | --- |
| GT | game-related measures | eye movement improvement scores |  |  |
|  | Game grades | The percentage of total duration on threatening stimuli changes | 0.244 | 0.422 |
|  | Total playing time | The percentage of total duration on threatening stimuli changes | 0.249 | 0.351 |
|  | Total game playing behaviors | The percentage of total duration on threatening stimuli changes | 0.403 | 0.122 |
|  | Active degree | The percentage of total duration on threatening stimuli changes | -0.213 | 0.485 |
|  | Game grades | The percentage of total fixations on threatening stimuli changes | -0.275 | 0.363 |
|  | Total playing time | The percentage of total fixations on threatening stimuli changes | 0.115 | 0.673 |
|  | Total game playing behaviors | The percentage of total fixations on threatening stimuli changes | 0.224 | 0.404 |
|  | Active degree | The percentage of total fixations on threatening stimuli changes | -0.516 | 0.071 |
|  | Game grades | The percentage of total duration on happy stimuli changes | 0.244 | 0.422 |
|  | Total playing time | The percentage of total duration on happy stimuli changes | 0.022 | 0.934 |
|  | Total game playing behaviors | The percentage of total duration on happy stimuli changes | 0.044 | 0.872 |
|  | Active degree | The percentage of total duration on happy stimuli changes | -0.213 | 0.485 |
|  | Game grades | The percentage of total fixations on happy stimuli changes | 0.453 | 0.120 |
|  | Total playing time | The percentage of total fixations on happy stimuli changes | 0.295 | 0.268 |
|  | Total game playing behaviors | The percentage of total fixations on happy stimuli changes | 0.401 | 0.124 |
|  | Active degree | The percentage of total fixations on happy stimuli changes | 0.253 | 0.404 |
|  | PANSS | The percentage of total duration on threatening stimuli changes | 0. 172 | 0. 541 |
|  | BNSS | The percentage of total duration on threatening stimuli changes | -0. 304 | 0. 270 |
|  | PSP | The percentage of total duration on threatening stimuli changes | -0. 234 | 0. 442 |
|  | TEPS | The percentage of total duration on threatening stimuli changes | -0. 309 | 0. 282 |
|  | SES | The percentage of total duration on threatening stimuli changes | -0. 332 | 0. 227 |
|  | SQLS | The percentage of total duration on threatening stimuli changes | 0. 287 | 0. 319 |
|  | MCCB | The percentage of total duration on threatening stimuli changes | -0. 082 | 0. 822 |
|  | PANSS | The percentage of total fixations on threatening stimuli changes | 0. 054 | 0. 848 |
|  | BNSS | The percentage of total fixations on threatening stimuli changes | -0. 491 | 0. 063 |
|  | PSP | The percentage of total fixations on threatening stimuli changes | -0. 001 | 0. 997 |
|  | TEPS | The percentage of total fixations on threatening stimuli changes | -0. 035 | 0. 904 |
|  | SES | The percentage of total fixations on threatening stimuli changes | -0. 153 | 0. 586 |
|  | SQLS | The percentage of total fixations on threatening stimuli changes | -0. 202 | 0. 489 |
|  | MCCB | The percentage of total fixations on threatening stimuli changes | -0. 023 | 0. 950 |
|  | PANSS | The percentage of total duration on happy stimuli changes | 0. 125 | 0. 657 |
|  | BNSS | The percentage of total duration on happy stimuli changes | -0. 127 | 0. 653 |
|  | PSP | The percentage of total duration on happy stimuli changes | -0. 190 | 0. 534 |
|  | TEPS | The percentage of total duration on happy stimuli changes | 0. 188 | 0. 521 |
|  | SES | The percentage of total duration on happy stimuli changes | 0. 203 | 0. 469 |
|  | SQLS | The percentage of total duration on happy stimuli changes | -0. 079 | 0. 787 |
|  | MCCB | The percentage of total duration on happy stimuli changes | 0. 319 | 0. 368 |
|  | BNSS | The percentage of total fixations on happy stimuli changes | 0. 119 | 0. 674 |
|  | PSP | The percentage of total fixations on happy stimuli changes | -0. 375 | 0. 206 |
|  | TEPS | The percentage of total fixations on happy stimuli changes | -0. 245 | 0. 398 |
|  | SES | The percentage of total fixations on happy stimuli changes | -0. 202 | 0. 470 |
|  | SQLS | The percentage of total fixations on happy stimuli changes | 0. 342 | 0. 231 |
|  | MCCB | The percentage of total fixations on happy stimuli changes | 0. 315 | 0. 375 |

Note: Abbreviations: GT, game training; TAU, treatment as usual; PANSS, positive and negative syndrome scale; BNSS, brief negative symptoms scale; PSP, personal and social performance scale; TEPS, temporal experience of pleasure scale; SES, self-esteem scale; SQLS, schizophrenia quality of life scale; MCCB, measurement and treatment research to improve cognition schizophrenia (MATRICS) consensus cognitive battery.

Table S15 A list of Komori Life examples of exercise

| **Exercise** | **Description** |
| --- | --- |
| **Memory** |  |
| Remember your neighbors | Memorize new locations and name pairs |
| **Attention** |  |
| Catch animals | Accurately click on the target animal images to capture it |
| **Executive function** |  |
| Maze map | Find your way out of a maze garden |
| **Language** |  |
| Decorate the house | Name your house, pets, and decorations |
| **Affect perception** |  |
| Match the feeling | Choose the face that expresses the same emotion as the neighbor’ face |
| **Social Cue Perception** |  |
| Flashback | Memorize a sequence of faces |
| **Social interaction** |  |
| Social community | Communicate with your neighbors and be familiar with your community |
| Collaboration and teamwork | Collaborate with your team members towards a common goal, such as capturing large animals |


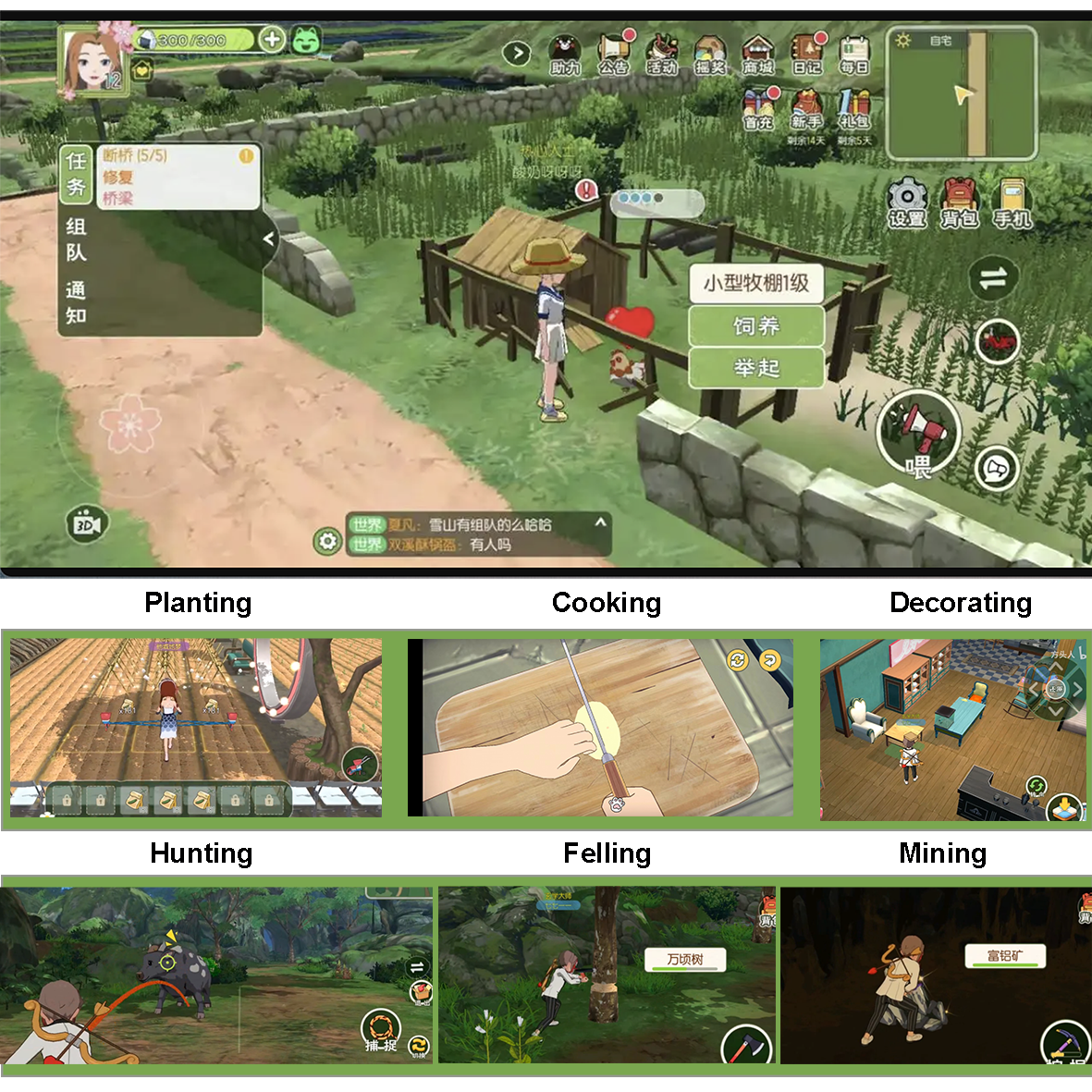


Figure S1 Screenshot from the Komori Life Training Program
